# MicroRNA bta-miR-365-3p inhibits proliferation but promotes differentiation of primary bovine myoblasts by targeting the activin A receptor type I

**DOI:** 10.1186/s40104-020-00528-0

**Published:** 2021-01-12

**Authors:** Dan Hao, Xiaogang Wang, Xiao Wang, Bo Thomsen, Yu Yang, Xianyong Lan, Yongzhen Huang, Hong Chen

**Affiliations:** 1 College of Animal Science and Technology, Northwest A&F University, Shaanxi Key Laboratory of Animal Genetics, Breeding and Reproduction, Yangling, 712100 Shaanxi China; 2 Department of Molecular Biology and Genetics, Aarhus University, 8000 Aarhus C, Denmark; 3 Quantitative Genomics, Bioinformatics and Computational Biology Group, Department of Applied Mathematics and Computer Science, Technical University of Denmark, Richard Petersens Plads, Building 324, 2800 Kongens Lyngby, Denmark

**Keywords:** *ACVR1*, Bta-miR-365-3p, Cattle, Primary bovine myoblast

## Abstract

**Background:**

MicroRNAs act as post-transcriptional regulators that repress translation or degrade mRNA transcripts. Each microRNA has many mRNA targets and each mRNA may be targeted by several microRNAs. Skeletal muscles express a plethora of microRNA genes that regulate muscle development and function by controlling the expression of protein-coding target genes. To expand our understanding of the role of microRNA, specifically bta-miR-365-3p, in muscle biology, we investigated its functions in regulating primary bovine myoblast proliferation and differentiation.

**Results:**

Firstly, we found that bta-miR-365-3p was predominantly expressed in skeletal muscle and heart tissue in Chinese Qinchuan beef cattle. Quantitative PCR and western blotting results showed that overexpression of bta-miR-365-3p significantly reduced the expression levels of cyclin D1 (*CCND1*), cyclin dependent kinase 2 (*CDK2*) and proliferating cell nuclear antigen (*PCNA*) but stimulated the expression levels of muscle differentiation markers, i.e., *MYOD1*, *MYOG* at both mRNA and protein level. Moreover, downregulation of bta-miR-365-3p increased the expression of *CCND1*, *CDK2* and *PCNA* but decreased the expression of *MYOD1* and *MYOG* at both mRNA and protein levels. Furthermore, flow cytometry, EdU proliferation assays and immunostaining results showed that increased levels of bta-miR-365-3p suppressed cell proliferation but promoted myotube formation, whereas decreased levels of bta-miR-365-3p resulted in the opposite consequences. Finally, we identified that activin A receptor type I (*ACVR1*) could be a direct target of bta-miR-365-3p. It was demonstrated that bta-miR-365-3p can bind to the 3’UTR of *ACVR1* gene to regulate its expression based on dual luciferase gene reporter assays. Consistently, knock-down of *ACVR1* was associated with decreased expressions of *CDK2*, *CCND1* and *PCNA* but increased expression of *MYOG* and *MYOD1* both at mRNA and protein level.

**Conclusion:**

Collectively, these data suggested that bta-miR-365-3p represses proliferation but promotes differentiation of bovine myoblasts through several biological mechanisms involving downregulation of *ACVR1*.

**Supplementary Information:**

The online version contains supplementary material available at 10.1186/s40104-020-00528-0.

## Background

Skeletal muscles originate from embryonic structures called somites, where mononuclear myoblasts proliferate, differentiate and fuse to multinucleated myotubes and subsequently differentiate into myofibers [1]. Myofibers vary with respect to their myosin heavy chain isoforms (fast versus slow) and types of energy metabolism (oxidative versus glycolytic). The number of myofibers is constant, but myofibers can increase in size by fusion with muscle stem cells, the satellite cells [2]. For example, adult skeletal muscle has a remarkable ability to repair after injury, leading to new myofiber formation in this process that involves satellite cells [3]. In addition, skeletal muscle mass and muscle fiber characteristics play key roles in the determination of meat yield and quality in cattle. Therefore, understanding the molecular processes and genetic networks underlying myogenesis and muscle development will provide fundamental information for cattle breeding programs.

The mature microRNAs (miRNAs) are small RNA molecules (~ 22 nucleotides), which have been widely identified in humans and animals since they were firstly discovered in the nematode *Caenorhabditis elegans* [4]. MicroRNAs act as post-transcriptional regulators that repress translation or degrade mRNA transcripts through either complete (canonical sites) or incomplete (non-canonical sites) complementarity with the 3’UTR of target mRNAs. Currently, the sequencing technologies are accelerating the discovery of microRNAs. Meanwhile, effective microRNA target sites are accurately predicted using various computational approaches [5]. The latest database of miRbase (Release 22.1) contains 48,860 distinct mature miRNA (miR) sequences from 271 organisms, including 1143 miRNAs from cattle [6].

Previous studies have revealed that tissue-specific and developmental stage-specific miRNAs play critical functional roles in diverse cellular, physiological and developmental processes [7–9]. For example, muscle-specific miRNAs such as miR-1, miR-133 and miR-206 participates in ontogenesis and skeletal myogenesis through a modulation of muscle differentiation genes [10–12]. Profiling of miRNA expression patterns among eleven different tissues from beef cattle showed that bta-miR-365-3p was ubiquitously expressed but with the highest expression level in muscle, which suggested its regulatory role in muscle tissue [13]. Furthermore, bta-miR-365-3p was differentially expressed between fast- and slow-type muscles (semitendinosus versus masseter) in Japanese Black steers [14]. Moreover, 2.6 fold higher expression of bta-miR-365-3p was found in the adult stage compared to the fetal stage of muscle tissues in Qinchuan cattle. A similar tendency has been observed with well-known muscle-specific miRNA bta-miR-1, but opposite with bta-miR-206 [15] (Fig. S1A). Remarkable, the total number of sequence reads of bta-miR-365-3p in the proliferation stages were almost 3.5 times higher than in the differentiation stages of skeletal muscle-derived satellite cells in Chinese Simmental calves. Similar results were also found in bta-miR-378a-3p and bta-miR-23a [16].

Based on this, we speculate that bta-miR-365-3p probably plays an important role in muscle tissue development. Notably, it has been reported that miR-365-3p negatively regulated histone deacetylase 4 (*HDAC4*) to stimulate primary chondrocyte proliferation and differentiation in mouse and chicken [17] and to contribute to osteoarthritis pathogenesis in humans [18]. However, few studies reported the characterizations of the target genes and the regulatory network of bta-miR-365-3p in muscle cells. Thus, the objective of our study is to assess the expression level of bta-miR-365-3p in various bovine tissues and to investigate its influences on proliferation and differentiation of primary myoblast.

## Materials and methods

### Animal and cell culture

All animal experiments were approved by the Animal Care Commission of the College of Veterinary Medicine of Northwest A&F University (Permit Number: NWAFAC1019). Six tissue samples, i.e., heart, liver, spleen, lung, kidney, *longissimus dorsi* muscle, were collected from sixty-day-old fetuses (*n* = 3) and two-year-old adults (*n* = 3) of the Chinese Qinchuan (QC) beef cattle breed. All samples were provided by Shanxi Kingbull Livestock Co., Ltd., Baoji city, China. Primary bovine myoblasts (PBMs) were isolated from fetal *longissimus dorsi* muscle following the established protocols [19]. Myoblasts were cultured in growth medium (GM) consisting of high-glucose Dulbecco’s modified Eagle’s medium (DMEM, Gibco) with 1% penicillin-streptomycin (HyClone) and 20% fetal bovine serum (TransGen, Beijing, China). Myoblast differentiation was stimulated in DMEM containing 2% horse serum (HyClone) and 1% penicillin-streptomycin (differentiation medium, DM). Cells were incubated at 37 °C with 5% CO_2_.

### Plasmid construction and transfection

A DNA fragment containing the precursor sequence of bta-miR-365-3p was obtained from genomic DNA of QC cattle by PCR and was inserted into the pcDNA-3.1(+) vector using T4 DNA ligase (Takara, Dalian, China). The resulting plasmid was named OPmiR-365-3p and used for overexpression of bta-miR-365-3p in PBMs.

The sequence of bta-miR-365-3p inhibitor is AUAAGGAUUUUUAGGGGCAUUA. With a 21–23 nt 2′-methoxy modified RNA oligonucleotide design, the bta-miR-365-3p inhibitor is a purified molecules that specifically and effectively inhibit endogenous mature bta-miR-365-3p’s activities. The sequence of the inhibitor’s negative control is CAGUACUUUUGUGUAGUACAA, which acted as the control group for the bta-miR-365-3p inhibitor treatment group (Table S1).

A DNA fragment containing the target site of bta-miR-365-3p in the 3’UTR of bovine *AVCR1* was amplified by PCR and cloned into the *XhoI* and *NotI* sites of the psiCHECK-2 dual-luciferase reporter vector (Promega, Madison, WI, USA) and the construct was named ACVR1-wild. Mutagenic primers were used to mutagenize the bta-miR-365-3p target site, which was cloned into psiCHECK-2 to create ACVR1-mutant. Three siRNAs of *ACVR1* were used to inhibit the expression of *ACVR1* in PBMs, including siACVR1-1, siACVR1-2 and siACVR1-3. The sequence of the siACVR1s is shown in Table S1.

Cells were transfected with OPmiR-365-3p, the inhibitor of bta-miR-365-3p, inhibitor N. C, ACVR1-wild, ACVR1-mutant and siACVR1s using Lipofectamine 2000 (Invitrogen, Grand Island, NY, USA) and incubated with 5% CO_2_ at 37 °C. The inhibitors and siRNAs were purchased from GenePharma (Shanghai, China). All experiments were performed in triplicate. All primers, inhibitors and the siRNAs sequences are listed in Table S1.

### RNA extraction and qRT-PCR

Total RNA was extracted from six different tissues and from PBMs using TRIzol reagent (Takara, Japan). After assessing RNA purity and concentration by spectrophotometry using a NanoDrop 2000 (Wilmington, USA) and 0.8% agarose gel electrophoresis, 1000 ng RNAs were transcribed into complementary DNA (cDNA) with PrimeScript RT reagent kit for use in qRT-PCR with SYBR Green Master Mix Reagen kit (GenStar, Beijing, China). The specific stem-loop of bta-miR-365-3p was used to synthesize the first cDNA. All primers are listed in Table S1. The method of 2^−ΔΔCt^ was used to calculate the relative expression levels.

### Western blot analysis

All proteins were extracted from PBMs at 4 °C using the radioimmunoprecipitation assay lysis buffer (RIPA buffer) and phenylmethylsulfnoyl fluoride (PMSF) (Solarbio, Beijing, China). Proteins were measured and adjusted by using the BCA protein assay kit (MULTI SCIENCE, China) and denatured with 5× SDS loading buffer (Beyotime) at 98 °C for 10 min. The prepared proteins were separated by SDS-polyacrylamide gel electrophoresis and then transferred to polyvinylidene fluoride membranes. After being blocked with 5% skim milk solution, membranes were incubated with the specific primary antibodies and the secondary antibody. We visualized the membranes using ChemiDoc™ XRS+ system (Bio-Rad Laboratories) and ECL Plus reagents (Solarbio, Beijing, China). The primary antibodies including anti-CDK2 and anti-PCNA were obtained from Sangon Biotech (Shanghai, China). Here, anti-ACVR1, anti-cyclin D1, anti-MyoD and anti-MyoG were purchased from Abcam (Cambrige, MA, USA). Anti-β-actin were purchased from SungenBio (Tianjin, China). HRP-conjugated Goat Anti-Rabbit IgG was obtained from BBI Life Science (Shanghai, China). All the primary antibodies were diluted with primary antibody dilution buffer that was obtained from Beyotime (Haimen, China). Image Lab™ Software 6.0.1 was used to calculate the grayscale value of the proteins.

### EdU and flow cytometry assay

After the transfection of PBMs with the expression vectors, inhibitor and siRNAs, we employed the EdU proliferation assay to measure their influences on DNA synthesis using the Cell Light EdU DNA cell proliferation kit according to the instructions (RiboBio, Guangzhou, China). The stained cells were detected and calculated by fluorescence microscopy (DM5000B, Leica Microsystems). Cell cycle phases were assessed by a cell cycle testing kit (Multisciences, Hangzhou, China) on a flow cytometry instrument (FACS Canto II, BD Biosciences, USA). Briefly, the cells were seeded in 6-well plates and transfected for 24 h after the cells reached 60% confluence. Cold 70% ethanol was used to fix the harvested cells. After staining with 100 μg/mL of the PI master mix at 37 °C for 30 min, the cell suspension was subjected to flow cytometry.

### Immunofluorescence staining

After inducing PBMs differentiation for 4 d, 4% paraformaldehyde in PBS was used to fix differentiated myoblast in a plate for 20 min. 0.5% of Triton-X-100 was added to permeabilize the fixed myoblast for 10 min and the cells were blocked with 5% bovine serum albumin solution (BSA) at 4 °C for 2 h. Subsequently, we incubated primary antibody (anti-MyHC diluted 1:250; Abcam, Cambridge, MA, USA) at 4 °C overnight and incubated the corresponding fluorescent secondary antibody at 4 °C for 2.5 h. Finally, the cell nuclei were stained with DAPI and the images were captured by fluorescence microscope (DM5000B, Leica Microsystems, Germany). The degree of differentiation was measured by the fusion index that was calculated as the number of nuclei in the myotube and as a percentage of the total nuclei.

### Dual-luciferase reporter assay

Dual-luciferase reporter assay was applied to test the interaction of bta-miR-365-3p with its predicted targets. HEK293T cells were co-transfected with OPmiR-365-3p vector (or the empty vector) and other vectors containing ACVR1-wild or ACVR1-mutant. The dual-luciferase activity was analyzed on an MPPC luminescence analyzer (HAMAMATSU, Beijing, China) using the luciferase reporter assay kit (Promega, Madison, WI, USA) according to the manufacturer’s instructions. The results were calculated as the ratios of firefly to Renilla luciferase activities in three independent replicates.

### Bioinformatics analysis

The online databases TargetScan (http://www.targetscan.org/vert_72/) and miRmap (https://mirmap.ezlab.org/) were used to search for the targets of bta-miR-365-3p [20, 21]. VENNY tool (version 2.1) (https://bioinfogp.cnb.csic.es/tools/venny/index.html) was used to obtain the common targets from the two databases [22]. The R package *clusterProfiler* [23] was used to cluster the enrichments of Gene Ontology (GO) and Kyoto Encyclopaedia of Genes and Genomes (KEGG) for the common genes.

### Statistical analysis

All the quantitative data were presented as the mean ± SD. Each group has three independent experiments. Student’ *t-*test procedure was used to analyze the statistical significance between groups were analyzed by SPSS v19.0. In this study, one asterisk, two asterisks and three asterisks indicated *P* < 0.05, *P* < 0.01 and *P* < 0.001 between groups, respectively.

## Results

### Bta-miR-365-3p expression in cattle tissue and PBMs

**Fig. 1:**
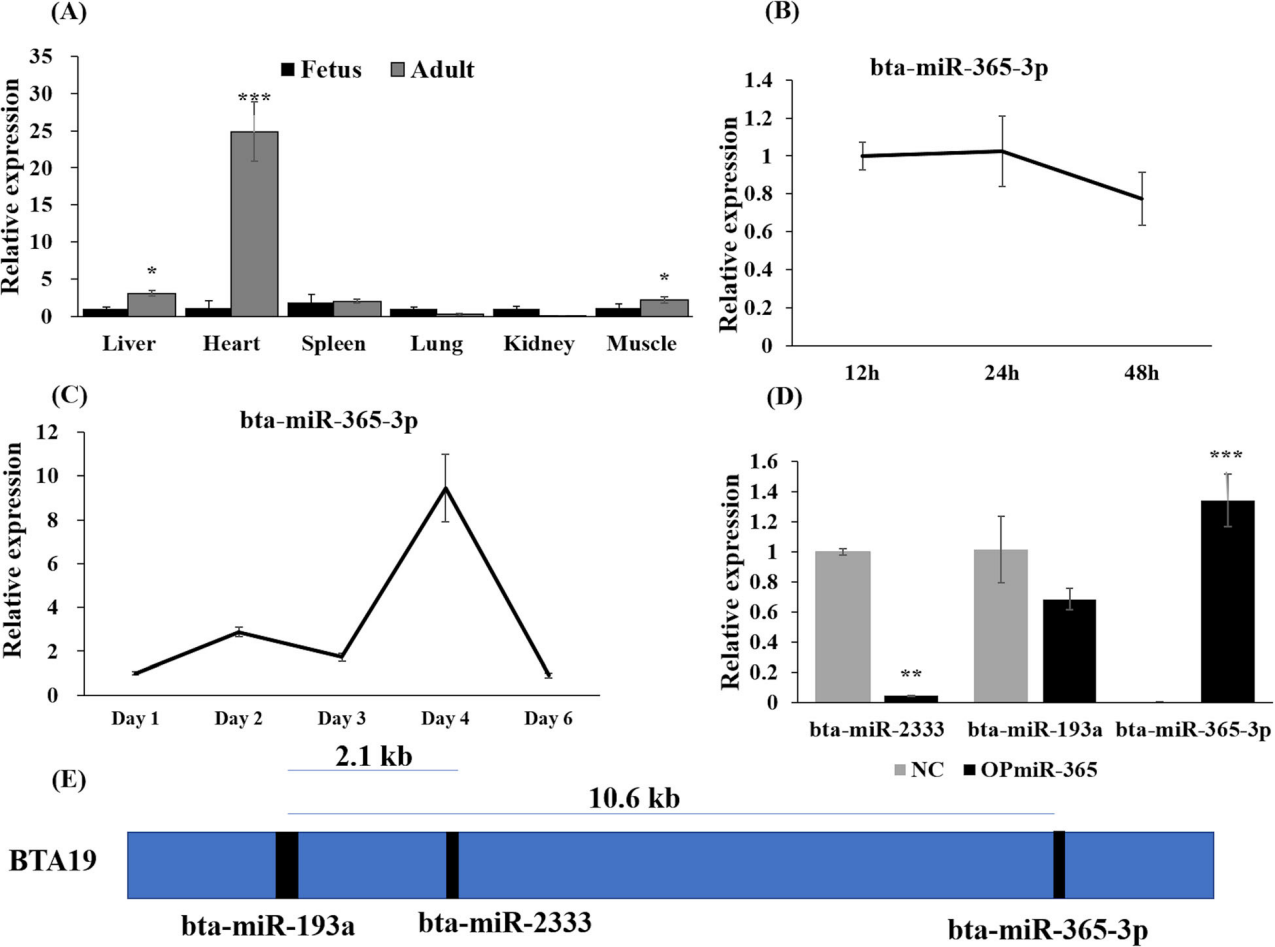
The expression level of bta-miR-365-3p at different development stages of cattle tissues and PBMs. **a** Differential expression level of bta-miR-365-3p in different tissues between fetal stage and adult stage of QC cattle. **b** bta-miR-365-3p expression levels during PBMs proliferation. **c** bta-miR-365-3p expression levels during PBMs differentiation with no treatment. **d** The expression levels of bta-miR-2333, bta-miR-193a and bta-miR-365-3p in PBMs, when transfected with OPmiR-365-3p in PBMs for 24 h in proliferation stage. NC, negative control with transfecting empty plasmid pcDNA 3.1. Note: The expression level of bta-miR-365-3p showed in the histogram was 100 times smaller. **e** The location of bta-miR-365-3p and its neighbor miRNAs in the bovine chromosome BTA19. All data are shown as mean ± SD for three biological replicates, the error bars indicated the SD among three repeats. Note:PBMs: primary bovine myoblasts; OPmiR-365-3p: the constructed pcDNA3.1 vector overexpressing bta-miR-365-3p; SD: standard deviation. *P < 0.05, **P < 0.01, *** *P* < 0.001

In order to investigate the functional roles of bta-miR-365-3p, we firstly detected the expression levels of bta-miR-365-3p in six different tissues of two developmental stages in QC cattle using quantitative PCR. The results showed that muscle and spleen tissues had the higher expression level in the fetus, while the expression of bta-miR-365-3p was highest in heart tissue of adult stage (Fig. 1a). Additionally, the expression levels of bta-miR-365-3p were significantly different (*P*-value < 0.05) between adult and fetal stages in the liver, heart and muscle tissues (Fig. 1a). We also found that the expression levels of bta-miR-365-3p exhibited a slightly decreased trend during PBM proliferation (Fig. 1b), while a dynamic expression profile that peaked on day four was observed during differentiation and subsequently rapidly decreased on day 6 (Fig. 1c). In the cultured myoblast cells transfected with the expression vector OPmiR-365-3p, quantitative PCR results showed that bta-miR-365-3p was significantly overexpressed, whereas the expression levels of bta-miR-2333 and bta-miR-193a that map close to bta-miR-365-3p on *Bos taurus* autosome (BTA) 19 were not significantly overexpressed (Fig. 1d and e), which indicated that the expression vector was constructed successfully.

### Bta-miR-365-3p suppresses PBM proliferation

**Fig. 2:**
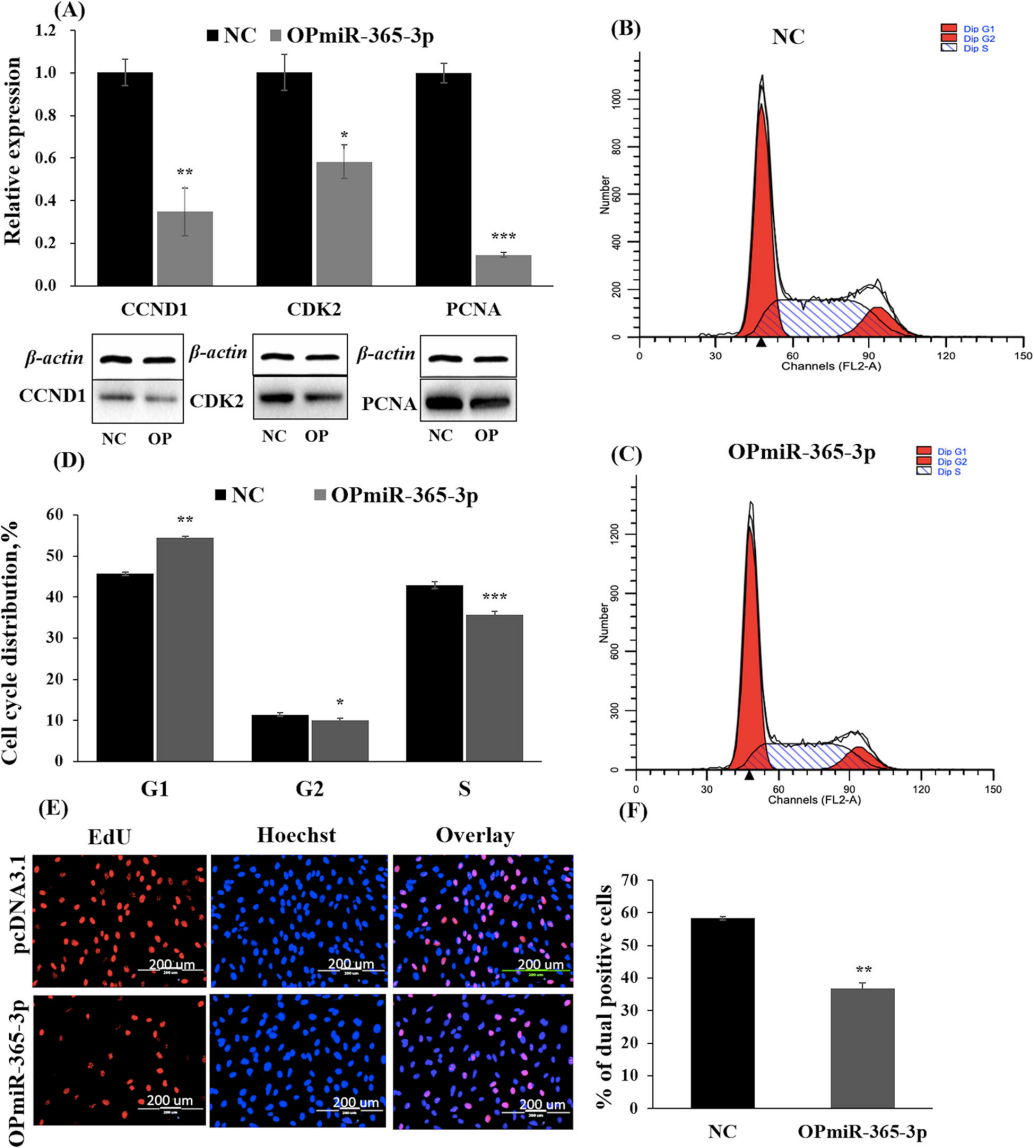
Transfection with OPmiR-365-3p and pcDNA.3.1 for 24 h in PBMs for detecting effects on cell proliferation. **a** The expression of *CDK2*, *PCNA* and *CCND1* at the mRNA level and protein level by qRT-PCR and western blotting, respectively. The OP group denotes the group treated with the vector OPmiR-365-3p, which overexpresses bta-miR-365-3p. **b** The PBMs transfected with empty plasmid pcDNA3.1 by flow cytometer assay. **c** The PBMs transfected with OPmiR-365-3p by flow cytometer assay. **d** Statistics of the counts of G_1_, G_2_ and S stage cells of flow cytometer assay. Error bars indicates the SD among three repeats. **e** EdU cell proliferation index, EdU (red), DAPI (blue), scale bars 200 μm. **f** Statistics of the percentage of dual positive cells. Error bars indicates the SD among three repeats. Values are mean ± SD for three biological replicates. Note: *CDK2*: cyclin-dependent kinase 2; *PCNA*: Proliferating cell nuclear antigen; *CCND1*: Cyclin D1 NC: Negative control transfected with the empty plasmid pcDNA3.1; OP: OPmiR_365-3p, the vector overexpressing bta-miR-365-3p; qRT-PCR: quantitative real-time polymerase chain reaction; SD: standard deviation. *P < 0.05, ** *P* < 0.01, *** *P* < 0.001

**Fig. 3:**
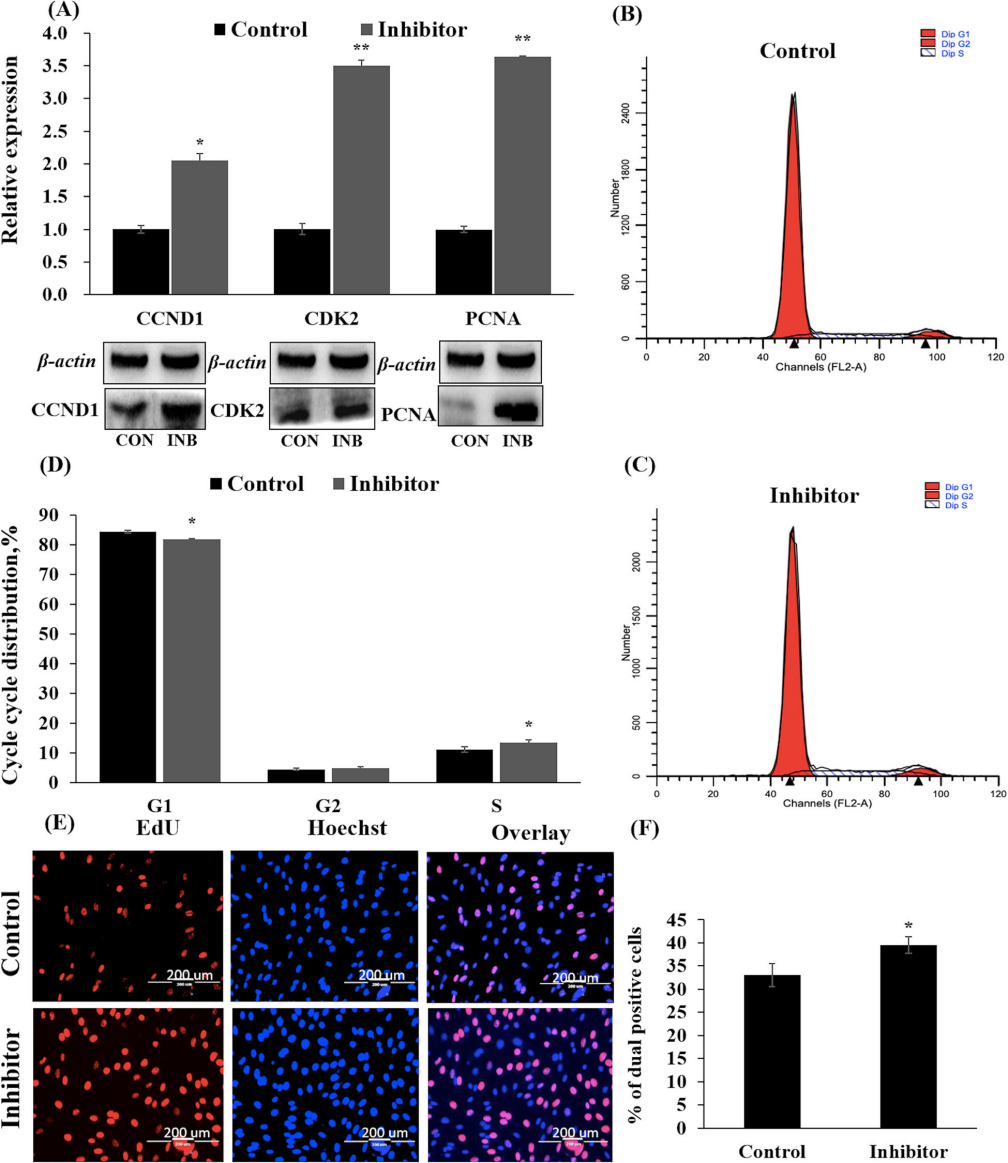
Transfection with the inhibitor of bta-miR-365-3p for 24 h in PBMs for detecting effects on cell proliferation. **a** The expression of *CDK2*, *PCNA* and *CCND1* at the mRNA level and protein level by qRT-PCR and western blotting, respectively. The CON group indicates the group treated with NC, and the INB group indicates the group treated with the inhibitor of bta-mR-365-3p. **b** The PBMs transfected with NC by flow cytometer assay. **c** The PBMs transfected with the inhibitor of bta-miR-365-3p by flow cytometer assay. **d** Statistics of the counts of G_1_, G_2_ and S stage cells of flow cytometer assay. **e** EdU cell proliferation index. EdU (red), DAPI (blue), scale bars 200 μm. **f** Statistics of the percentage of dual positive cells. Values are mean ± SD for three biological replicates. Note: *CDK2*: cyclin-dependent kinase 2; *PCNA*: Proliferating cell nuclear antigen; *CCND1*: Cyclin D1 NC: Negative control. qRT-PCR: quantitative real-time polymerase chain reaction; SD: standard deviation. *P < 0.05, ** *P* < 0.01

The proteins encoded by *CDK2* (cyclin-dependent kinase 2*)*, *PCNA* (proliferating cell nuclear antigen) and *CCND1* (cyclin D1) have been identified to perform critical functions in G_1_, S and G_2_ phases during cell cycle progression [24–26]. The results of qRT-PCR and western blotting showed that the expressions of *CCND1*, *CDK2* and *PCNA* were significantly decreased at both the mRNA and protein levels after transfecting PBMs with OPmiR-365-3p (Fig. 2a). Flow cytometer assays showed that PBM numbers were lower in the G_2_-phase (12.78%) and in the S-phase (16.91%) (*P* < 0.05 and *P* = 0.08, respectively), whereas the proportion of PBMs was increased in the G_0_/G_1_-phase, when bta-miR-365-3p was overexpressed (Fig. 2b, c and d). The EdU proliferation assays revealed a 36.86% reduction in mitotic activity of PMBs after transfection with OPmiR-365-3p (*P* < 0.01) (Fig. 2e and f). However, inhibition of bta-miR-365-3p significantly increased the expressions of the proliferation marker genes *CCND1*, *CDK2* and *PCNA* at both mRNA and protein levels (Fig. 3a). The proportion of PBMs was increased 20.06% in S-phase (*P* < 0.05), and decreased 3.1% in G_0_/G_1_-phase after we knocked down bta-miR-365-3p in PBMs (Fig. 3b, c and d). In addition, the number of EdU-positive cells increased 15.4% in the bta-miR-365-3p inhibitor group (Fig. 3e and f). Therefore, we suggested that the overexpression of bta-miR-365-3p suppressed PBM proliferation, while knockdown of bta-miR-365-3p promoted PBM proliferation.

### Bta-miR-365-3p promotes PBMs differentiation

**Fig. 4:**
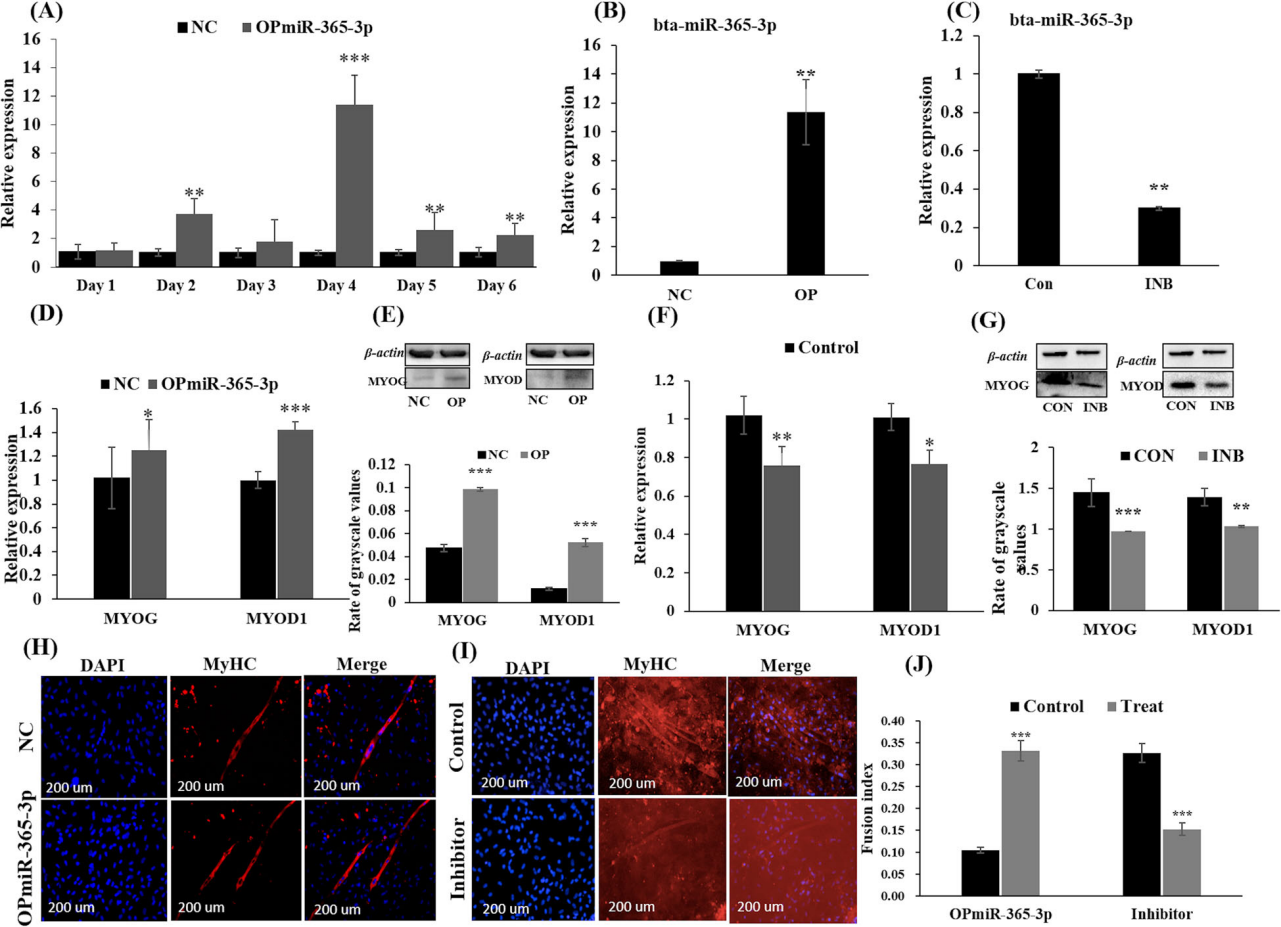
Overexpression of bta-miR-365-3p promoted primary cattle myoblast differentiation. **a** The expression level of miR-365-3p during PBMs differentiation after treatment with OPmiR-365-3p for differentiation six days. **b** The expression level of bta-miR-365-3p after treatment with OPmiR-365-3p in PBMs at four days. **c** The expression level of bta-miR-365-3p after treatment with its inhibitor for four days. **d** The mRNA expression of *MYOD1* and *MYOG* in PBMs after treatment with OPmiR-365-3p for four days. **e** The protein expression of MYOG and MYOD1 under the treatment of OPmiR-365-3p, and the statistic relatively rate grayscale values of MYOG or MYOD1 compared withβ-actin with Image Lab software. **f** The mRNA expression of MYOD1 and MYOG in PBMs after treatment with the inhibitor of bta-miR-365-3p for four days. **g** The protein expression of MYOG and MYOD1 under the treatment of bta-miR-365-3p inhibitor, and the statistic the relatively rate grayscale values of MYOG and MYOD1 compared withβ-actin with Image Lab software. **h** MyHC (red)-positive myotubes were detected by immunofluorescence after transfection with OPmiR-365-3p, scale bars: 200 μm. **i** MyHC (red)-positive myotubes were detected by immunofluorescence after transfection with the inhibitor of bta-miR-365-3p. Scale bars: 200 μm. **j** The fusion indexes calculation, the number of nuclei in the myotube as a percentage of the total nucleus. The ‘Treat’ indicated that the groups were treated with OPmiR-365-3p or the inhibitor. The ‘Control’ indicated the groups of their negative control. Values are mean ± SD for three biological replicates. Note: MYOD1: Myogenic differentiation 1; MYOG: Myogenin. SD: standard deviation. *P < 0.05, ** *P* < 0.01, *** *P* < 0.001

To understand the function of bta-miR-365-3p for PBMs differentiation, we monitored its expression levels following induction of differentiation and overexpression by continuously transfecting with OPmiR-365-3p. Expectedly, it also showed a peak expression level of bta-miR-365-3p during differentiation on day 4 (Fig. 4a). Subsequently, the effect of bta-miR-365-3p on PBM differentiation was assessed by an approximately 10-fold overexpressing after the transfection with OPmiR-365-3p (Fig. 4b), or 5-fold reduction of expression levels using an inhibitor of bta-miR-365-3p on differentiation day 4 (Fig. 4c). Both mRNA and protein expression levels of two muscle differentiation marker genes (i.e., *MYOD1* and *MYOG*) increased by OPmiR-365-3p (Fig. 4d and e), but decreased by bta-miR-365-3p inhibitor (Fig. 4f and g). Immunofluorescence staining results showed that bta-miR-365-3p overexpression gave a higher amount of MyHC-positive myotubes compared to the control group (Fig. 4h and j), while the opposite result was observed in the treatment with the bta-miR-365-3p inhibitor (Fig. 4i and j). Therefore, we suggested that bta-miR-365-3p promoted PBM differentiation.

### *ACVR1* is a target gene of the bta-miR-365-3p

**Fig. 5:**
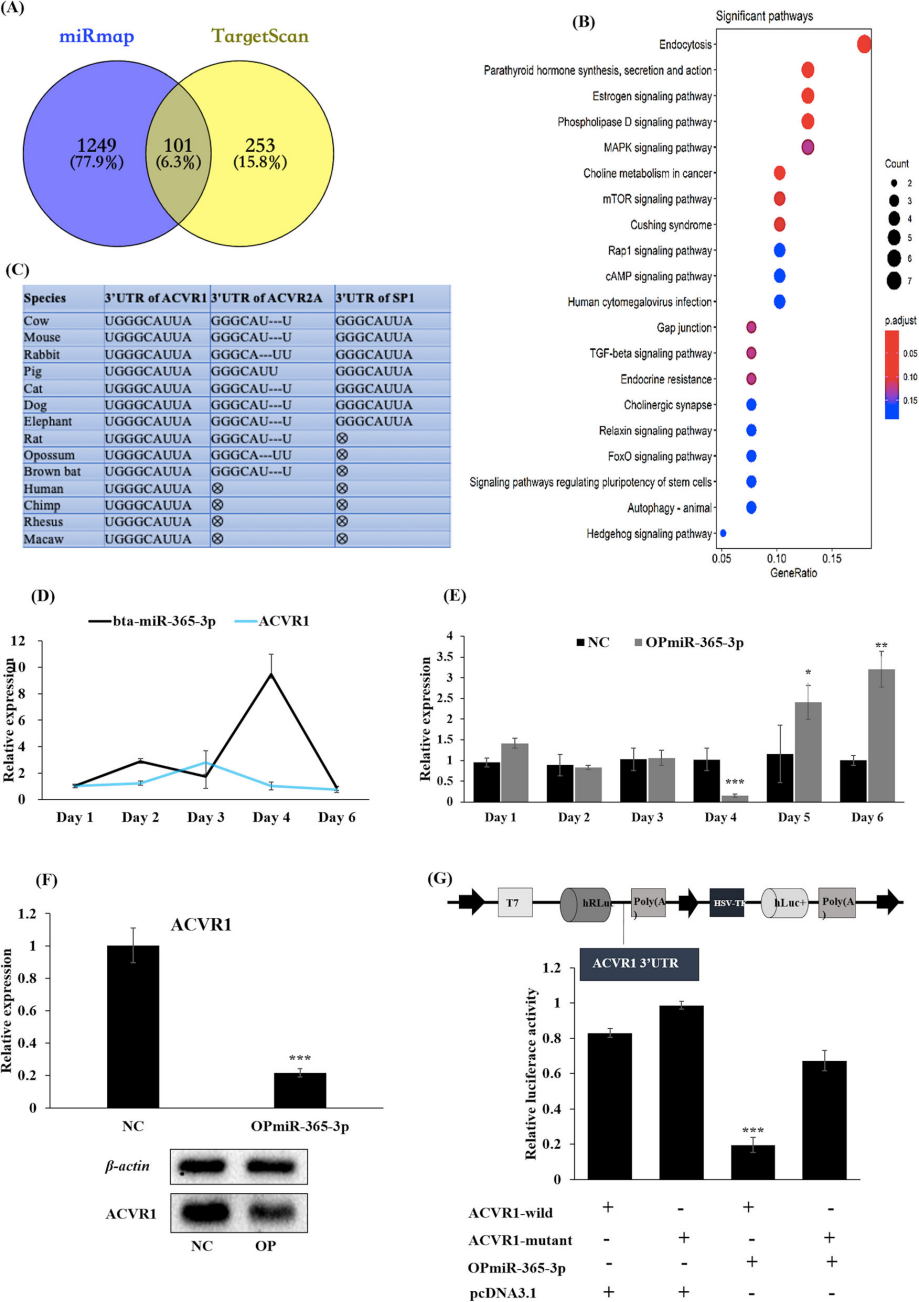
Bovine *ACVR1* is a target gene for miR-365-3p. **a** The common target genes of bta-miR-365-3p from two databases, miRmap and TargetScan. **b** The KEGG pathways associated with the common target genes of bta-miR-365-3p. The red to blue color indicated that p.adjust values was increasing, and the red circle means the genes were enriched in the significant pathway; the size of the circle indicates the gene counts in the pathway. **c** Conservation among various species of the bta-miR-365-3p target sequences in the 3′UTR of *ACVR1*, *ACVR2A*, *SP1.* **d** The expression level of *ACVR1* during PBMs differentiation. **e** The expression level of *ACVR1* after transfection with OPmiR-365-3p for continuously inducing differentiation 6 days. **f** The mRNA and protein expression of *ACVR1* when transfected with the interference siACVR1-1 cultured with growth medium (GM) for proliferating 24 h. **g** The structure of the psiCHECK-2 dual-luciferase reporter vector, and the luciferase activity test when transfected with ACVR1- wild, ACVR1-mutant, OPmiR-365-3p and pcDNA3.1(NC). Note: *ACVR1*: activin A receptor type I; ACVR2A: activin A receptor type 2A; Sp1: transcription factor; ACVR1- wild: psiCHECK-2 dual-luciferase reporter vector of the ACVR1 3′UTR region. ACVR1-mutant: psiCHECK-2 dual-luciferase reporter vector of the ACVR1 3′UTR region with mutation; SD: standard deviation. *P < 0.05, ** *P* < 0.01, *** *P* < 0.001

In silico prediction, TargetScan and miRmap revealed 1354 and 354 putative target genes of bta-miR-365-3p, respectively. The intersection of the predicted targets gave 101 genes, which were used in a KEGG pathway analysis (Fig. 5a). The five most significant signaling pathways (adjusted *P*-value < 0.05) were parathyroid hormone synthesis, secretion and action (bta04928), endocytosis (bta04144), estrogen signaling pathway (bta04915), phospholipase D signaling pathway (bta04072) and choline metabolism in cancer (bta05231) (Fig. 5b). Interestingly, the putative target genes of bta-miR-365-3p, *ACVR2A* (activin A receptor type 2A), *SP1* (Sp1 transcription factor) and *ACVR1* (activin A receptor type 1) were enriched in TGF-beta signaling pathway (bta04350) (*P-*value < 0.1) (Table S2). Moreover, the presence of target sites in the 3′ UTR of these three genes were analyzed in fourteen different animal species, which showed a strong conservation of the bta-miR-365-3p target sites, especially in *ACVR1* (Fig. 5c). The Fig. 5d showed that the expression level of *ACVR1* was inhibited when the endogenous bta-miR-365-3p was highly expressed on day 2 and especially on day 4. By contrast, the expression level of *ACVR1* showed higher expression when the endogenous bta-miR-365-3p was downregulated on day 3 (Fig. 5d). Furthermore, expression of *ACVR1* was significantly downregulated on day 4 after transfection with OPmiR-365-3p in PBMs (Fig. 5e). Consistently, overexpression of bta-miR-365-3p negatively regulated the *ACVR1* mRNA and protein levels in PBMs on day 4 (Fig. 5e and Fig. 5f). We also conducted a dual-luciferase activity experiment to test the interaction of bta-miR-365-3p with the 3′UTR of *ACVR1*. The data showed that the luciferase activity was significantly decreased when co-transfecting the OPmiR-365-3p and ACVR-wild vectors in PBMs. In contrast, the luciferase activity was unaffected in co-transfections of OPmiR-365-3p and ACVR1-mutant. Collectively, these results suggested that *ACVR1* could be a direct target gene for bta-miR-365-3p (Fig. 5g).

### siACVR1 inhibited PBM proliferation but promoted PBM differentiation

**Fig. 6:**
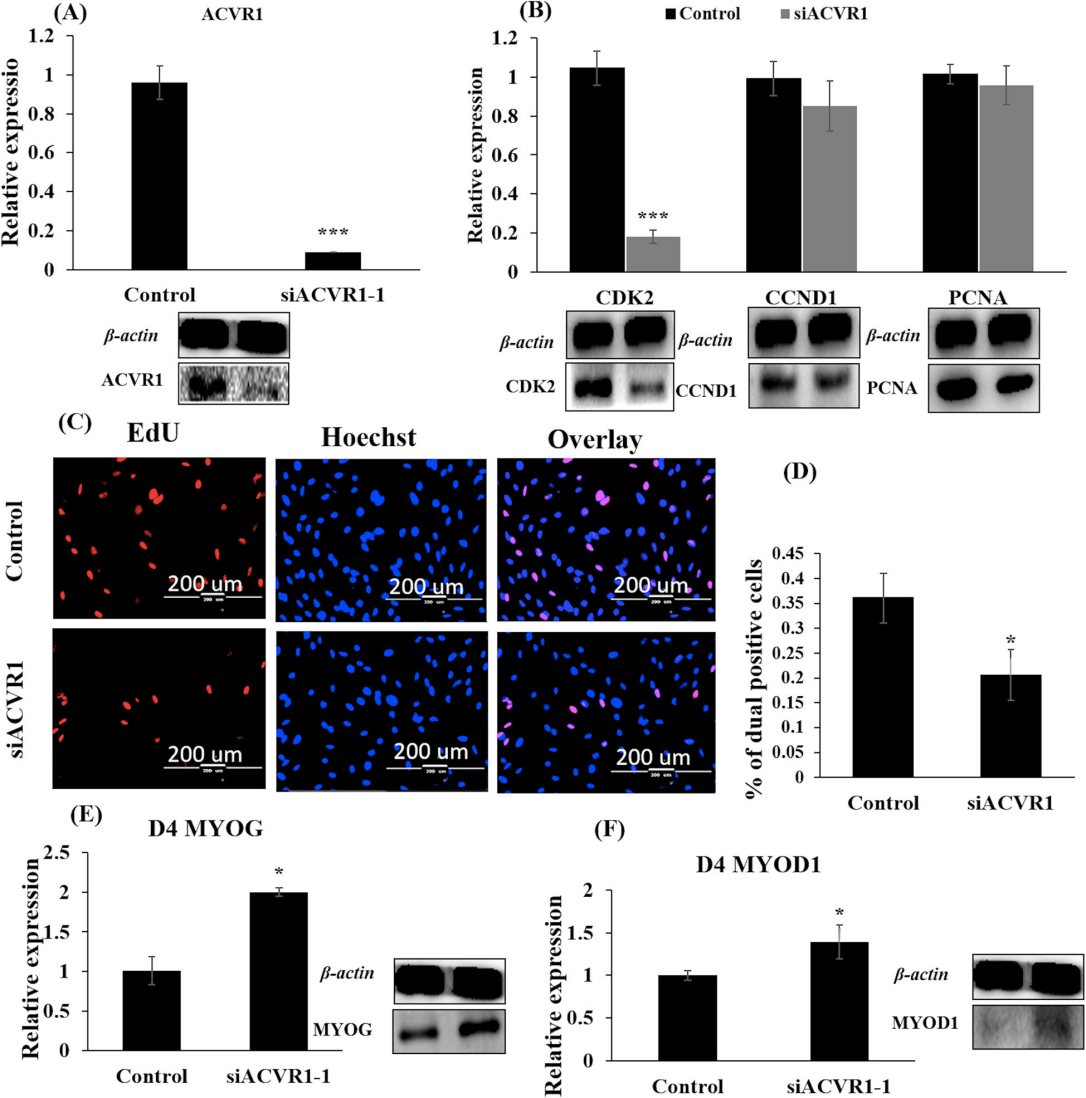
Knock-down of *ACVR1* inhibited primary myoblast proliferation but promoted PBMs differentiation. **a** RNA interference using siACVR1-1 decreases expression of *ACVR1* at mRNA and protein levels compared with the control group by qRT-PCR and western blotting in PBMs, respectively. **b** The mRNA and protein expression levels of *CDK2*, *CCND1*, and *PCNA* at 24 h. post transfection with siACVR1-1 in PBMs. **c** EdU proliferation assay detected the cell proliferation index. EdU (red), Hoechst (blue), scale bars 2000 μm. **d** Statistics of the percentage of dual positive cells. Error bars indicates the SD among three repeats. **e** The mRNA and protein expression of *MYOG* in PBMs after treated with siACVR1-1 and induced differentiation for four days. **f** The mRNA and protein expression of *MYOD1* in PBMs after treated with siACVR1-1 induced for four days. Values are mean ± SD. Note: CDK2: cyclin-dependent kinase 2; PCNA: Proliferating cell nuclear antigen; MYOD1: Myogenic differentiation 1; MYOG: Myogenin; siACVR1: The interference RNA of *ACVR1* in cattle; SD: standard deviation. *P < 0.05; *** *P* < 0.001

Next, we employed siRNA technology to address the role of *ACVR1* in PBM. Three different siRNAs were designed to target the bovine *ACVR1* and to be transfected in PBMs. It showed that siACVR1-1 downregulated the expression of *ACVR1* efficiently at both the mRNA and protein levels in PBMs (Fig. 6a). Furthermore, the analysis of q-RT-PCR and western blotting found that siACVR1-1 also significantly decreased *CDK2* expression at both mRNA and protein levels. *CCND1* and *PCNA* were only slightly decreased at mRNA level but significantly reduced at the protein level (Fig. 6b). In the EdU proliferation assay, the percentage of dual positive PBMs was significantly lower in cells after knock-down of *ACVR1* than in non-treated cells (Fig. 6c and Fig. 6d). Conversely, the muscle differentiation markers *MYOD1* and *MYOG* were increased at both mRNA and protein levels after knock-down of *ACVR1* in PBMs (Fig. 6e and f). Therefore, overexpression of bta-miR-365-3p and knock-down of *ACVR1* expression have similar consequences on PBMs proliferation and differentiation.

## Discussion

### miRNAs in bovine skeletal muscle

Skeletal muscle mass and muscle fiber characteristics are highly related to important economical traits such as meat quality and yield in beef cattle [27]. Understanding the molecular genetics of bovine skeletal muscle development will, therefore, provide important information for using in cattle breeding programs. Recently, advanced sequencing and bioinformatics technologies as well as the annotated databases like miRbase have revealed several miRNAs associated with bovine skeletal muscle development and differentiation of satellite cells [6, 13–16, 28]. MiRNAs regulate protein synthesis by targeting mRNAs; so far four microRNAs modulating bovine skeletal muscle development and function have been deposited in miRTarBase, which provides information about experimentally validated miRNA-target interactions [29]. Six miRNAs regulating proliferation, apoptosis and differentiation of PBMs through targeting of various functional genes have been confirmed by various experimental methods [30–35] (Table 1). For example, miR-744 was abundantly expressed in fetal stage of Qinchuan cattle and has been confirmed to positively regulate proliferation of skeletal muscle satellite cells [15, 31].

**Table 1:** The published miRNAs to skeletal muscle myoblast development in cattle

miRNAs name	Target gene	Target ID	Associated phenotype	References PMID
bta-miR-1	HDAC4	517,559	Skeletal muscle satellite cell myogenic differentiation	26424132
bta-miR-206	HDAC4	517,559	Skeletal muscle satellite cell myogenic differentiation	26424132
bta-miR-1	LOC536229	536,229	Skeletal muscle satellite cell myogenic differentiation	26424132
bta-miR-206	LOC536229	536,229	Skeletal muscle satellite cell myogenic differentiation	26424132
bta-miR-23a	ZNF423	508,025	Adipogeneses in skeletal muscle	28255176
bta-miR-27b	MSTN	281,187	Skeletal muscle hypertrophy	23510267
bta-miR-208b	CDKN1A	513,497	Promoted skeletal muscle cell proliferation	30317561
bta-miR-744	Wnt5a	530,005	Promoted skeletal muscle cell proliferation while inhibited the apoptosis and differentiation	31051333
bta-miR-744	CaMKIIδ	109,560,236	Promoted skeletal muscle cell proliferation while inhibited the apoptosis and differentiation	31051333
bta-miR-148a-3p	KLF6	505,884	Inhibited muscle cell proliferation but promoted apoptosis	30793769
bta-miR-378a-3p	HDAC4	517,559	Promoted myoblast differentiation and inhibited proliferation	27661135
bta-miR-125b	IGF2	281,240	Sponged by lncMD to promote myoblast differentiation	27589905
bta-miR-107	Wnt3a	522,467	Suppress cell differentiation and did not affect cell proliferation	29858062
bta-miR-885	MYOD1	281,938	Promote proliferation but inhibit differentiation	331985035

### The expression profile and functional roles of bta-miR-365-3p

Previous studies have shown that the expression levels of bta-miR-365-3p were significantly differently expressed in the fast- and slow-type skeletal muscles in the stages of myoblast differentiation and the different developmental stages of cattle [13–16], supporting its functional roles in skeletal muscle development. Skeletal muscle development can be divided into the prenatal stage that decides the muscle fibers numbers and the postnatal stage that mainly generates the muscle fiber size [36]. Firstly, we demonstrated that bta-miR-365-3p was highly expressed in heart and skeletal muscle tissues in Qinchuan cattle (Fig. 1a), which was consistent with the previous transcriptome study in various tissues of Angus crossbred cattle [13]. Subsequently, bta-miR-365-3p had higher expression in adult than in fetal stages of muscle tissues that was similar to bta-miR-1 in Sun et al. [15] (Fig. S1A). Thus, the results potentially indicate more important roles of bta-miR-365-3p in the postnatal stage development of skeletal muscle to affect the fiber size than in the prenatal stage development to affect the muscle fibers numbers.

The dynamic process of myoblast developed to myofiber involved proliferation, determination, differentiation and maturation phases [37, 38]. In our study, endogenous bta-miR-365-3p showed higher expression in the maturation stage (72 h–96 h, myotubes to form myofiber) than in the early differentiation stage (24 h–72 h, mononucleated fuse to multinucleated myotubes). The same expression tendency was found in the specific-related skeletal muscle development miRNAs, such as bta-miR-1 and bta-miR-23a but not bta-miR-125b in a previous study (Fig. S1B) [16]. In the early myoblast differentiation, bta-miR-1 expression was higher than bta-miR-365-3p, suggesting that bta-miR-1 is more important role for the early differentiation stage than bta-miR-365-3p. In agreement, we observed that the endogenous bta-miR-365-3p plays an important role in the maturation stage of the myoblast differentiation process of rather than in early myoblast differentiation stage [16] (Fig. 1c). The lower expressed after 4 days differentiation, indicated that once the myofibers were fused, the function of bta-miR-365-3p may be reduced.

It has been reported that miR-365-3p served as a therapeutic biomarker for various cancers and tumors such as lung cancer [39, 40], colon cancer [28], pancreatic cancer [41], breast cancer [42] and gastric tumorigenesis [43]. Moreover, miR-365-3p inhibited vascular smooth muscle cell proliferation through the targeting of *CCND1* [44]. This agrees well with the present study, demonstrating that bta-miR-365-3p acted as a negative regulator of PBM proliferation. Thus, cell cycle analysis with high levels of bta-miR-365-3p showed an increased the percentage of cells in the G_0_/G_1_-phase and reduced the number of cells in S-phase, whereas, downregulated bta-miR-365-3p with its inhibitor showed opposite effects. Consistently, *CDK2*, *CCND1* and *PCNA* were all shown to be downregulated when overexpressing bta-miR-365-3p, while these marker genes were upregulated when the expression of bta-miR-365-3p was inhibited in PBMs. In contrast, overexpression of bta-miR-365-3p increased the expressions of muscle differentiation markers (*MYOD1*, *MYOG*) and promoted myoblast differentiation and myotube formation, while inhibition of bta-miR-365-3p showed the reverse effects. These observations are similar to previous results, showing that miR-365-3p promoted chondrocyte differentiation [18].

### Gene targets of miR-365-3p

Several targets of miR-365-3p have been validated in other studies such as cyclin D1 (*CCND1*) [44, 45], histone deacetylase 4 (*HDAC4*) [17, 18], nuclear factor I/B (*NFIB*) [46], Pax 6 [47] thyroid transcription factor 1 (*TTF1*) [39], src homology domain containing 1 (SHC1) and Bax [41] (Table 2). After bioinformatics analysis, we identified that *ACVR1*, also known as ALK2, a member of bone morphogenetic protein receptors type I, was another target of bta-miR-365-3p. As an essential member of TGF-β family, *ACVR1* has the functional roles in early embryonic development [48], lens formation [49], chondrogenesis, osteogenesis [50, 51] and cardiac hypertrophy [52]. Additionally, recurrent heterozygous mutations of *ACVR1* were associated with diseases in human such as fibro dysplasia ossificans progress (FOP) [53], diffuse intrinsic pontine gliomas (DIPGs) [54] and pediatric midline high-grade astrocytoma (mHGAs) [55]. The mutations of *ACVR1* were also associated with meat weight, eye muscle area, silverside weight, and growth traits in cattle [56, 57]. Moreover, a constitutively activating mutation of *ACVR1* induced the expression of Tmem176b in C2C12 cells and promoted myoblast differentiation into osteoblasts [58]. In our study, we found that *ACVR1* is a direct target of bta-miR-365-3p, and the decreased expression of *ACVR1* significantly inhibited myoblast proliferation but promoted myoblast differentiation. Our study was consistent to Shi et al. research, who used the antisense oligonucleotides (AONs) to knockdown *ACVR1* expression in mouse, which also resulted in the induction of muscle differentiation and repression of osteoblast differentiation [59].

**Table 2:** The published functions of miR-365-3p and its validated targets

Target genes	Genes name	Functions	References PMID
HDAC4	Histone deacetylase 4	Stimulate chondrocyte differentiation in chicken or mouse/osteoarthritis development in human	21856783
HDAC4	Histone deacetylase 4	Osteoarthiritis development in human	27023516
TTF1	Thyroid transcription factor1	Regulate lung cancer	22185756 and 26337230
CycD1/Bcl2	Cyclin D1/Bcl apoptosis regulator 2	Regulate colon cancer	22072615
SHC1	Src homology domain containing 1	Gemcitabine Regulate pancreatic cancer	24216611
NFIB	Nuclear factor I/B	Promote cutaneous squamous cell carcinoma	24949940
CycD1/cdc25A	Cyclin D1	Contribute to gastric tumorigenesis	24149576
CycD1	Cyclin D1	Inhibit vascular smooth muscle cell proliferation	24819721 and 24936138
Pax6	Paired box 6	Regulate human retinoblastoma cells	23660406
Not clearly		Transport-related stress in turkeys	26760121
Kcnh2	Potassium voltage-gated channel subfamily H member 2	Regulate nociceptive behaviors	26937014
IL-6	Interleukin-6	Host defense	21518763
Bcl-2	Bcl apoptosis regulator 2	Response low-density lipoprotein stimulation	21640710

## Conclusions

In summary, we found that bta-miR-365-3p was predominantly expressed in muscle tissues from adult and fetal stages. It also repressed the proliferation but promoted the differentiation of PBMs through the downregulation of *ACVR1* in cattle.

## Supplementary Information

**Additional file 1: Table S1** The primers used in this study. **Table S2** The pathway of the common target genes of bta-miR-365-3p from two database. **Figure S1** The expression level of previously identified miRNAs. (A) The fold change (FC) values between adult stage of muscle tissues and fetal stage of muscle tissues in Qinchuan cattle based on the Sun et al’s study [15]. (B) The FC values among primary muscle cell proliferation stage (P), primary muscle cell differentiation stage for 1 day (D1) and primary muscle cell differentiation stage for 3 days (D3) based on Zhang et al’s study [16]. D1/P indicated the FC values between D1 and P. D3/P indicated the FC values between D3 and P. D3/D1 indicated the FC values between D3 and D1. All the FC calculation is based on $\frac{\mathrm{A}\ \mathrm{reads}-\mathrm{B}\ \mathrm{reads}}{\min \kern0.5em \left(\mathrm{A}\ \mathrm{reads},\kern0.75em \mathrm{B}\ \mathrm{reads}\right)}$. **Figure S2** The expression level of *ACVR1* after transfected with siACVR1s.

## Data Availability

The data sets used and/or analyzed during the current study are available from the corresponding author on reasonable request.

## Abbreviations

ACVR1: Activin A receptor type I
CCND1: Cyclin D1; CDK2: Cyclin dependent kinase 2
DIPGs: Diffuse intrinsic pontine gliomas
DM: Differentiation medium
FOP: Sporadic fibro dysplasia ossificans progressive
GM: Proliferation medium
GO: Gene Ontology
HDAC4: Histone deacetylase 4
KEGG: Kyoto Encyclopaedia of Genes and Genomes
MYOD1: Myogenic differentiation 1
MYOG: Myogenin
myomiR: Muscle-specific miRNAs
mHGAs: Midline high-grade astrocytoma
NFIB: Nuclear factor I/B
PCNA: Proliferating cell nuclear antigen
PBM: Primary bovine myoblast
PMSF: Phenylmethylsulfnoyl fluoride
RIPA: Radioimmunoprecipitation assay buffer
SHC1: Src homology domain containing 1
TTF1: Thyroid transcription
