# Supplementary material for: MicroRNA bta-miR-365-3p inhibits proliferation but promotes differentiation of primary bovine myoblasts by targeting the activin A receptor type I
Source: J Anim Sci Biotechnol. 2021 Jan 12;12:16. doi: 10.1186/s40104-020-00528-0 (PMC7802253; doi:10.1186/s40104-020-00528-0)
Supplement: Supplementary file 1 — Additional file 1: Table S1 The primers used in this study. Table S2 The pathway of the common target genes of bta-miR-365-3p from two database. Figure S1 The expression level of previously identified miRNAs. (A) The fold change (FC) values between adult stage of muscle tissues and fetal stage of muscle tissues in Qinchuan cattle based on the Sun et al’s study [15]. (B) The FC values among primary muscle cell proliferation stage (P), primary muscle cell differentiation stage for 1 day (D1) and primary muscle cell differentiation stage for 3 days (D3) based on Zhang et al’s study [16]. D1/P indicated the FC values between D1 and P. D3/P indicated the FC values between D3 and P. D3/D1 indicated the FC values between D3 and D1. All the FC calculation is based on $\frac{\mathrm{A}\ \mathrm{reads}-\mathrm{B}\ \mathrm{reads}}{\min \kern0.5em \left(\mathrm{A}\ \mathrm{reads},\kern0.75em \mathrm{B}\ \mathrm{reads}\right)}$Areads−BreadsminAreadsBreads. Figure S2 The expression level of ACVR1 after transfected with siACVR1s. [file 40104_2020_528_MOESM1_ESM.docx]

**Table S1** The primers

| Gene | Sense | Antisense | Base pair |
| --- | --- | --- | --- |
| *PCNA* | TCCAGAACAAGAGTATAGC | TACAACAGCATCTCCAAT | 94 |
| *CCND1* | GACGAGCTGCTGCACATGGA | TGCTTGTTCTCCTCGGCCAC | 125 |
| *CDK2* | TTTGCTGAGATGGTGACCCG | TAACTCCTGGCCAAACCACC | 115 |
| *MYOD* | ACGGCATGATGGACTACAGC | AGGCAGTCGAGGCTCGACA | 133 |
| *MYOG* | AGGGGATCATCTGCTCCCAG | ATCCCGGCAGACAATCTCAG | 143 |
| *ACVR1* | CTGTGCTCGTGATGATTGCT | AGCTTGGGGTTTACCTTGGG | 70 |
| *GAPDH* | CACTGAGGACCAGGTTGTCT | TGTCGTACCAGGAAATGAGC | 119 |
| ACVR1-F | AATTCTAGGCGATCGCTCGAGTGGTGTCAAGAAGGAAGATC | | 290 |
| ACVR1-R | TATTTTATTGCGGCCAGCGGCCGTTCCTCCAGTTCCCTACCTT | |  |
| ACVR1-M-F1 | AATTCTAGGCGATCGCTCGAGTGGTGTCAAGAAGGAAGATC | | 222 |
| ACVR1-M-R1 | TATTTTATTGCGGCCAGCGGCCGGTGAAAACCCCAGGTCACAG | |  |
| ACVR1-M-F2 | AATTCTAGGCGATCGCTCGAGCTGTGACCTGGGGTTTTCAC | | 109 |
| ACVR1-M-R2  Bta-miR-365-3p-F-precure  Bta-miR-365-3p-R-precure  Inhibitor-bta-miR-365  Inhibitor N.C | TATTTTATTGCGGCCAGCGGCCGTTCCTCCAGTTCCCTACCTT  AACTTCCTATGGGCTGGGAG  ATCAGCAAATCCCCCTACAG  AUAAGGAUUUUUAGGGGCAUUA  CAGUACUUUUGUGUAGUACAA | |  |
| miR365-3P-RT | GTCGTATCCAGTGCAGGGTCCGAGGTATTCGCACTGGATACGACATAAGG | | |
| miR365-3P-F | TAATGCCCCTAAAAATCCT |  |  |
| miR365-3P-R | CAGTGCAGGGTCCGAGGTAT |  |  |
| siACVR1-178 | GGAUGGAGUGAUGAUUCUUTT | AAGAAUCAUCACUCCAUCCTT |  |
| siACVR1-658 | GGAAUAUGGCACCAUUGAATT | UUCAAUGGUGCCAUAUUCCTT |  |
| siACVR1-1109 | GCGAGCGGUCUCGCACAUUTT | AAUGUGCGAGACCGCUCGCTT |  |

Note: ACVR1-F and ACVR1-R were used to amplified the nonmutated 3’UTR of *ACVR1*; ACVR1-M-F1 and ACVR1-M-R1, ACVR1-M-F2 and ACVR1-M-R2 were used to amplified the mutated 3’UTR of *ACVR1*; the bta-miR-365-3p-F-precure and bta-miR-365-3p-R-precure were used to amplified the precursor sequence of bta-miR-365-3p. Inhibitor-bta-miR-365: the inhibitor of bta-miR-365-3p; Inhibitor N.C: the control of inhibitor bta-miR-365-3p; siACVR-178, siACVR1-658, siACVR1-1109 were used for the interference of ACVR1 in primary cattle muscle cells. Other primers were used for q-RT-PCR.

Table S2. The pathway of the common target genes of bta-miR-365-3p from two database

| ID | Description | GeneRatio | BgRatio | *P* value | *P*.adjust | *Q* value | geneID | Count |
| --- | --- | --- | --- | --- | --- | --- | --- | --- |
| bta04928 | Parathyroid hormone synthesis, secretion and action | 5/39 | 104/8654 | 9.47E-05 | 0.00687551 | 0.00559025 | 509936/281638/533199/540245/540741 | 5 |
| bta04144 | Endocytosis | 7/39 | 245/8654 | 9.48E-05 | 0.00687551 | 0.00559025 | 281638/508199/281831/282301/100125224/520739/511223 | 7 |
| bta04915 | Estrogen signaling pathway | 5/39 | 138/8654 | 0.00036 | 0.01725587 | 0.01403018 | 509936/535704/281831/533199/540741 | 5 |
| bta04072 | Phospholipase D signaling pathway | 5/39 | 152/8654 | 0.00056 | 0.02017596 | 0.01640441 | 509936/533199/282301/100125224/537312 | 5 |
| bta05231 | Choline metabolism in cancer | 4/39 | 99/8654 | 0.00097 | 0.02827371 | 0.02298843 | 533199/282301/100125224/540741 | 4 |
| bta04150 | mTOR signaling pathway | 4/39 | 154/8654 | 0.00489 | 0.10598271 | 0.08617106 | 533199/527459/515854/618601 | 4 |
| bta04934 | Cushing syndrome | 4/39 | 156/8654 | 0.00512 | 0.10598271 | 0.08617106 | 509936/533199/507449/540741 | 4 |
| bta04540 | Gap junction | 3/39 | 90/8654 | 0.00759 | 0.12396872 | 0.10079489 | 509936/533199/282301 | 3 |
| bta04350 | TGF-beta signaling pathway | 3/39 | 93/8654 | 0.0083 | 0.12396872 | 0.10079489 | 338068/281598/540741 | 3 |
| bta01522 | Endocrine resistance | 3/39 | 94/8654 | 0.00855 | 0.12396872 | 0.10079489 | 509936/533199/540741 | 3 |
| bta04010 | MAPK signaling pathway | 5/39 | 292/8654 | 0.0095 | 0.12527269 | 0.10185511 | 281638/281831/533199/282301/510245 | 5 |
| bta04725 | Cholinergic synapse | 3/39 | 114/8654 | 0.01442 | 0.1742334 | 0.14166345 | 509936/281883/533199 | 3 |
| bta04015 | Rap1 signaling pathway | 4/39 | 216/8654 | 0.01568 | 0.17487695 | 0.14218671 | 509936/533199/282301/537312 | 4 |
| bta04024 | cAMP signaling pathway | 4/39 | 229/8654 | 0.01903 | 0.18560013 | 0.15090537 | 509936/540830/533199/537312 | 4 |
| bta04926 | Relaxin signaling pathway | 3/39 | 130/8654 | 0.02042 | 0.18560013 | 0.15090537 | 509936/281638/533199 | 3 |
| bta04068 | FoxO signaling pathway | 3/39 | 131/8654 | 0.02084 | 0.18560013 | 0.15090537 | 533199/515854/504480 | 3 |
| bta04340 | Hedgehog signaling pathway | 2/39 | 51/8654 | 0.02196 | 0.18560013 | 0.15090537 | 281638/540830 | 2 |
| bta05163 | Human cytomegalovirus infection | 4/39 | 245/8654 | 0.02373 | 0.18560013 | 0.15090537 | 509936/533199/282301/540741 | 4 |
| bta04550 | Signaling pathways regulating pluripotency of stem cells | 3/39 | 139/8654 | 0.02432 | 0.18560013 | 0.15090537 | 338068/281598/533199 | 3 |
| bta04140 | Autophagy - animal | 3/39 | 142/8654 | 0.0257 | 0.18634768 | 0.15151318 | 538831/533199/618601 | 3 |

Figure S1. The expression level of previously identified miRNAs. (A) The fold change (FC) values between adult stage of muscle tissues and fetal stage of muscle tissues in Qinchuan cattle based on the Sun et al’s study [15]. (B) The FC values among primary muscle cell proliferation stage (P), primary muscle cell differentiation stage for 1 day (D1) and primary muscle cell differentiation stage for 3 days (D3) based on Zhang et al’s study [16]. D1/P indicated the FC values between D1 and P. D3/P indicated the FC values between D3 and P. D3/D1 indicated the FC values between D3 and D1. All the FC calculation is based on $\frac{A reads-B reads}{\min(A reads, B reads)}$.

Figure S2. The expression level of ACVR1 after transfected with siACVR1s.
